# Do Changes in Transtympanic Electrocochleography (TT-ECochG) Reflect the Dynamics of Endolymphatic Hydrops on MRI? Audiological and Radiological Longitudinal Analysis in Patients with Severe Ménière’s Disease After Vestibular Neurectomy—A Pilot Study

**DOI:** 10.3390/jcm15187164

**Published:** 2026-09-15

**Authors:** Agnieszka Jasinska-Nowacka, Emilia Wnuk, Izabela Pobożny, Achala Kamath, Kazimierz Niemczyk

**Affiliations:** 1 Department of Otorhinolaryngology, Head and Neck Surgery, Medical University of Warsaw, 02-091 Warsaw, Poland; izabela.pobozny@wum.edu.pl (I.P.); kamath.achala@gmail.com (A.K.); kazimierz.niemczyk@wum.edu.pl (K.N.); 2 2nd Department of Clinical Radiology, Medical University of Warsaw, 02-091 Warsaw, Poland; emilia.wnuk@wum.edu.pl; 3 Dr. Kirtane’s Clinic, Mumbai 400004, India

**Keywords:** Ménière’s disease, endolymphatic hydrops, vestibular neurectomy, electrocochleography, TT-ECochG

## Abstract

**Objectives:** The aim of this study was to evaluate long-term changes in transtympanic electrocochleography (TT-ECochG) following vestibular neurectomy in patients with Ménière’s disease (MD) and to investigate their relationship with endolymphatic hydrops (EH) degree and hearing outcomes. **Methods:** This pilot study included five patients with unilateral MD eligible from a cohort of twenty patients who underwent middle fossa vestibular neurectomy. All participants completed preoperative and 2-year postoperative clinical and audiological tests, including TT-ECochG; EH degree was analyzed in delayed gadolinium-enhanced magnetic resonance images (MRI). Changes in TT-ECochG parameters were analyzed in relation to MRI findings. **Results:** All patients achieved complete control of vertigo and improvement in the AAO-HNS functional level. Hearing deterioration of ≥10 dB was observed in two patients (40%). Cochlear EH remained unchanged in all patients, whereas vestibular EH reduction was visualized in two patients (40%) after surgery. Decreased SP/AP amplitude and/or area ratios were observed in two patients (40%), whereas increased values were found in two others (40%). No postoperative TT-ECochG response was obtained in one patient because of profound postoperative hearing loss. Overall, changes in TT-ECochG parameters did not parallel MRI findings. **Conclusions:** This pilot study demonstrates that postoperative TT-ECochG changes after vestibular neurectomy are heterogeneous and do not consistently parallel MRI changes in endolymphatic hydrops.

## 1. Introduction

Clinical manifestation of Ménière’s disease (MD) is characterized by recurrent attacks of spinning vertigo accompanied by tinnitus, aural fullness, and hearing loss [1,2,3,4,5,6]. According to the guidelines established by the American Academy of Otolaryngology-Head and Neck Surgery (AAO-HNS) [2] and the Barany Society [3], diagnosis of MD is based on clinical symptoms confirmed by pure-tone audiometry. Nevertheless, a variety of audiological tests are performed in patients with MD to evaluate inner ear function during the natural course of endolymphatic hydrops. According to the AAO-HNS guidelines, MD management follows a therapeutic escalation starting from diet, lifestyle modification, and conservative therapies and gradually transitioning into surgical methods [1,2,3,4,5].

Dynamic advancement of magnetic resonance imaging (MRI) methods in recent years has allowed in vivo confirmation of endolymphatic hydrops (EH), considered to be the morphological cause of MD [7,8,9,10]. Since then, numerous studies have analyzed correlations between the EH degree and audiovestibular test results [11,12]. Moreover, several studies described the dynamics of EH after conservative and surgical treatment [13,14,15,16,17,18,19].

Electrocochleography (ECochG) is an electrophysiological audiological examination that records the electrical activity of the cochlear cells and the cochlear nerve in response to acoustic stimuli [20,21,22,23,24,25,26,27,28,29]. The two most commonly used ECochG techniques differ according to electrode placement: the electrode may be positioned on the tympanic membrane (extratympanic electrocochleography, ET-ECochG) or directly on the cochlear promontory (transtympanic electrocochleography, TT-ECochG).

The value of the TT-ECochG in MD diagnostics was first reported in 1977 by Gibson et al. [20], who observed abnormal SP/AP waveforms in patients with certain MD diagnoses. Since then, the method has been included in the set of dedicated tests for the diagnosis of MD; however, the diagnostic value of ECochG is under discussion [21,26]. Various criteria and methods for evaluating results can be used—analysis of the summating potential to action potential amplitude ratio (SP/AP amp ratio), the ratio of the area under the curves of these potentials (SP/AP area ratio), and the difference in latency of the AP potential depending on the polarization (con-rar AP lat shift). Due to the diversity of technical details and results analysis, no established guidelines or gold standards exist for interpreting ECochG results [25,28,29].

ECochG is considered an indicator of endolymphatic hydrops; therefore, it can presumably serve as a practical method for evaluating inner ear condition after treatment.

In the present study, TT-ECochG results and EH degree were analyzed before and after vestibular neurectomy in patients with uncontrollable MD. The objective was to verify if electrocochleographic indicators of endolymphatic hydrops could change after vestibular denervation. Moreover, we aimed to describe the relationships among changes in TT-ECochG results, EH severity dynamics visualized on MRI, and the intensity of vestibular and aural symptoms of MD.

## 2. Materials and Methods

### 2.1. Patient Description and Clinical Evaluation

Twenty patients with unilateral definite MD according to the AAO-HNS guidelines [2] and Barany Society criteria [3], who underwent middle fossa vestibular neurectomy, were prospectively followed with clinical, audiological, and MRI examinations before surgery and at the 2-year follow-up. Due to the invasive character of the TT-ECochG procedure, only five patients agreed to undergo repeat examinations before and after the surgery. Therefore, only these five patients, for whom complete preoperative and postoperative data were available, were included in the present pilot study.

Patients self-assessed the severity of main clinical symptoms during the preoperative visit. The average levels of tinnitus, aural fullness, and balance problems during the interattack periods were rated on a 0–6 scale proposed by Arenberg and Stahle [30]. Patients reported the number of vertigo attacks within each of the last six months, and the average frequency of MD episodes was calculated. Dizziness handicap inventory (DHI) was used to evaluate patients’ disability [31]. Additionally, general functional level was self-assessed using a 1–6 scale established by the AAO-HNS [2]. At the postoperative visit, the same protocol was used to evaluate the clinical outcome. The follow-up interval ranged from 22 to 24 months.

### 2.2. Ethical Consideration

The study was approved by the University Ethics Committee (approval Nos.: KB/110/2019 and AKBE/65/2024).

### 2.3. Audiological Test Evaluation Protocol

Audiological tests were performed before and after vestibular neurectomy.

Pure-tone audiometry was performed in all patients. The pure-tone average PTA was calculated from air-conduction thresholds at 0.5, 1, 2, and 4 kHz. A postoperative increase in PTA exceeding 10 dB relative to the preoperative value was classified as clinically significant hearing deterioration [32]. TT-ECochG was conducted using the Smart Box device with Smart EP software (Intelligent Hearing Systems, FL, Miami, USA). After applying local anesthesia into the external auditory canal, a thin needle at the end of the electrode was inserted through the tympanic membrane into the tympanic cavity near the promontory.

The examination was performed monaurally with click stimuli at 90 dB nHL, presented at rates of 7.11/s and 49.9/s. Ipsilateral responses were saved in 3 blocks of 256 sweeps, and the mean result was subsequently assigned. Two methods were used in the analysis of the TT-ECochG results as follows:

1. The ratio of the summating potential amplitude to the action potential amplitude (SP/AP amp ratio) was calculated. An SP/AP ratio > 0.33 was considered abnormal, while values exceeding 0.5 were interpreted as strongly suggestive of endolymphatic hydrops [28].
2. The ratio of the surface areas of both potentials was analyzed (SP/AP area ratio). Values exceeding 1.94 were considered abnormal [29].

### 2.4. Magnetic Resonance Imaging

Magnetic resonance imaging (MRI) was performed preoperatively and repeated two years after vestibular neurectomy. Examinations were performed using a 3-T scanner (Signa Architect, GE Healthcare, Milwaukee, WI, USA) equipped with a 16-channel flex coil (GEM Flex Large coil, Neocoil, Pewaukee, WI, USA). The imaging protocol comprised 3D-FLAIR sequences acquired before and 4 h after intravenous administration of a double dose of gadobutrol (Gadovist, Bayer Schering Pharma AG, Berlin, Germany; 1.0 mmol/mL). The MRI methodology was described in detail in a previous study [10]. Endolymphatic hydrops was assessed separately in the cochlea and vestibule using the 0–2 Baráth [8] and 0–3 Bernaerts [9] grading systems, respectively. Cochlear contrast enhancement was determined by comparison with the contralateral asymptomatic ear [9].

### 2.5. Treatment Qualification and Surgical Procedure

The decision for surgical treatment was made individually for each patient, based on overall audiological status and clinical disease severity. Before surgical qualification, all patients had undergone at least six months of unsuccessful conservative treatment. Each patient had received betahistine (24 mg twice daily) and at least two courses of intratympanic steroid therapy (four injections per course). Medical refractory MD was defined as persistent vertigo substantially affecting daily functioning (at least 4 points in the AAO-HNS functional level scale and/or Tumarkin’s attacks). To avoid potential overlapping treatment effects, patients with a history of intratympanic gentamicin therapy and previous middle or inner ear surgery were excluded from the study. Patients aged ≤65 years with residual hearing considered worth preserving were considered eligible for selective vestibular nerve section via the middle fossa approach. All procedures were performed by the same highly experienced otologic surgeon. To minimize the risk of vestibular nerve regeneration, the vestibular ganglion was surgically removed. On the seventh or eighth day after surgery, once the acute vestibular symptoms had resolved, each patient was discharged home in good condition.

## 3. Results

### 3.1. Patients’ Characteristics

Five patients with unilateral definite MD (three in the left ear, two in the right ear) were enrolled in this study (Table 1). The population age ranged from 35 to 65 years (mean, 51 years). Disease duration ranged from 1 to 18 years (mean, 7 years), measured from the first MD symptom. All patients denied any aural symptoms in the contralateral ear.

### 3.2. Preoperative Clinical Data

Before the surgery, patients reported typical vertigo episodes with an average frequency of 3.9 attacks/month within the last six months. The patients self-assessed the severity of tinnitus, aural fullness, and balance problems during the interattack periods as 4.4, 3.53, and 3.77 on the 0–6 Arenberg scale, respectively. The DHI ranged from 72 to 100 points (mean 88), while the functional level, assessed on a 1–6 scale established by the AAO-HNS, was 5 for four patients and 1 for one patient. One patient (#3) reported Tumarkin’s drop attacks in his past medical history.

### 3.3. Postoperative Clinical Data

At follow-up, complete vertigo control was observed in all patients, with no attacks reported during the preceding six months (vertigo control class A according to AAO-HNS). The average severity of tinnitus, aural fullness, and balance problems decreased to 2.0, 1.1, and 2.2, respectively. The postoperative result in the DHI questionnaire ranged from 4 to 32 points and was lower in all patients (average 19.6). In the functional AAO-HNS scale, improvement was described in all five patients. Three patients were classified as functional level 2, whereas the remaining two patients achieved functional level 1.

### 3.4. Preoperative Audiological Results

**Figure 1:**
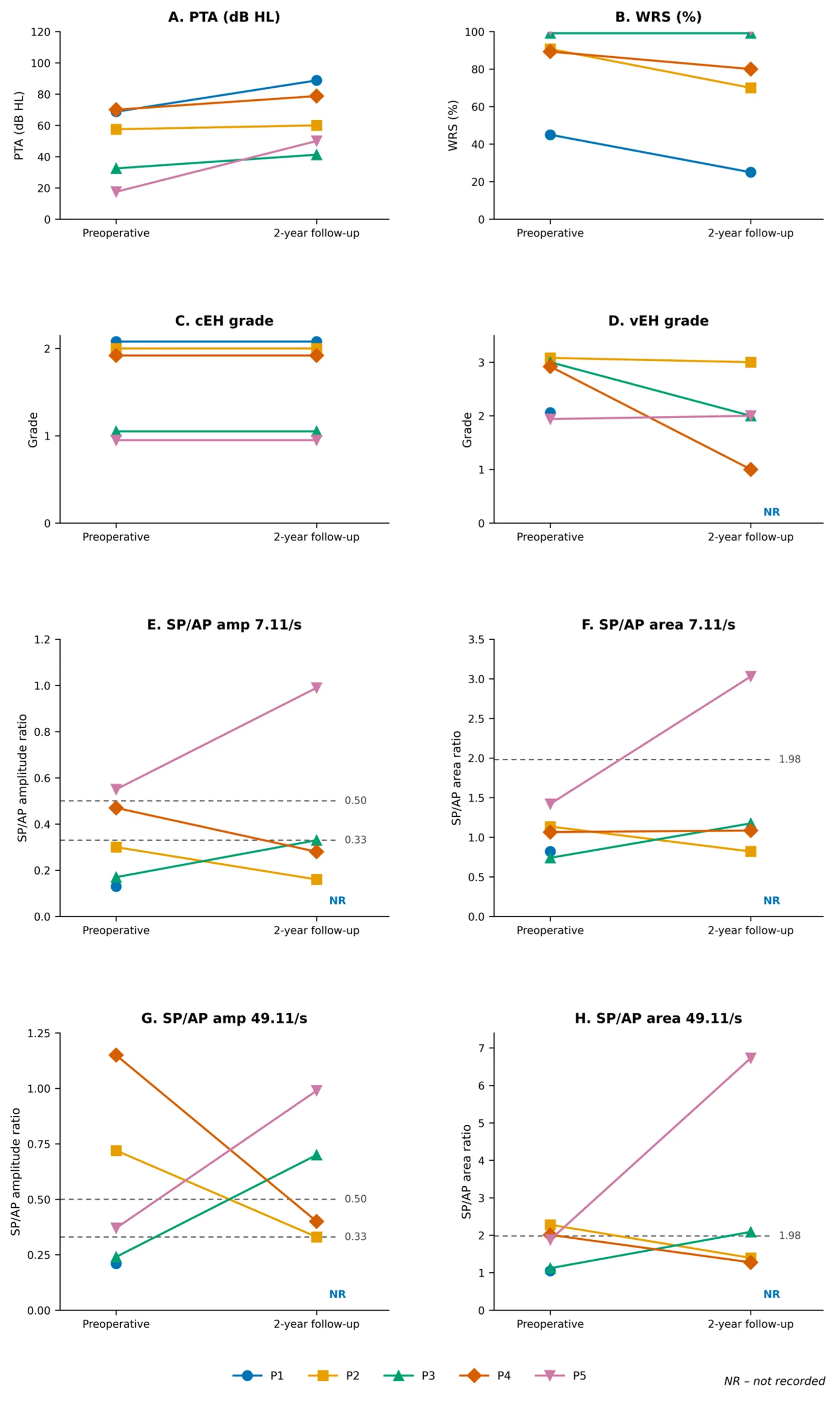
Long-term changes in hearing, endolymphatic hydrops degree, and transtympanic electrocochleography following vestibular neurectomy in patients with Ménière’s disease. Individual patient trajectories (*n* = 5) are shown for (**A**) pure-tone average (PTA; 0.5, 1, 2, and 4 kHz), (**B**) word recognition score (WRS), (**C**) cochlear endolymphatic hydrops (cEH, Baráth grading system), (**D**) vestibular endolymphatic hydrops (vEH, Bernaerts grading system), (**E**) SP/AP amplitude ratio at a stimulation rate of 7.11/s, (**F**) SP/AP area ratio at 7.11/s, (**G**) SP/AP amplitude ratio at 49.11/s, and (**H**) SP/AP area ratio at 49.11/s. NR indicates that postoperative examination could not be performed or interpreted properly.

**Figure 2:**
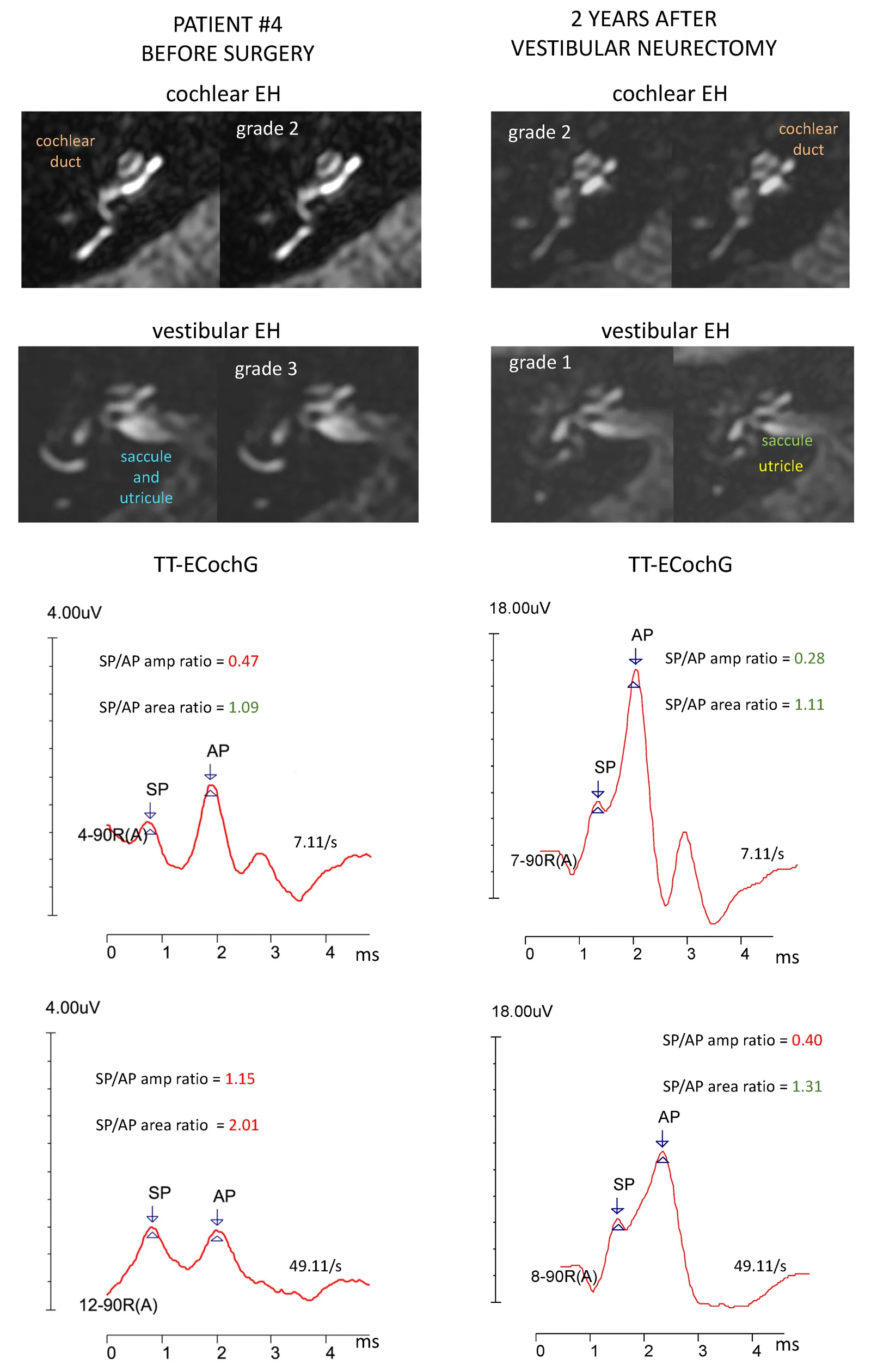
Representative changes in delayed gadolinium-enhanced MRI and transtympanic electrocochleography (TT-ECochG) following vestibular neurectomy. Individual results (patient #4) are shown before surgery (**left column**) and 2 years after vestibular neurectomy (**right column**). Upper panels demonstrate stable cochlear endolymphatic hydrops (cEH grade 2, Barath scale) and regression of vestibular endolymphatic hydrops (vEH grade 3 to grade 1, Bernaerts scale). To facilitate interpretation, line art of the endolymphatic structures is provided with the corresponding MRI images. The lower panels present TT-ECochG recordings obtained at stimulation rates of 7.11/s and 49.11/s. Abnormal results are highlighted in red and normal results in green. Following surgery, the SP/AP amplitude ratio at 7.11/s and both the SP/AP amplitude and area ratios at 49.11/s decreased.

**Figure 3:**
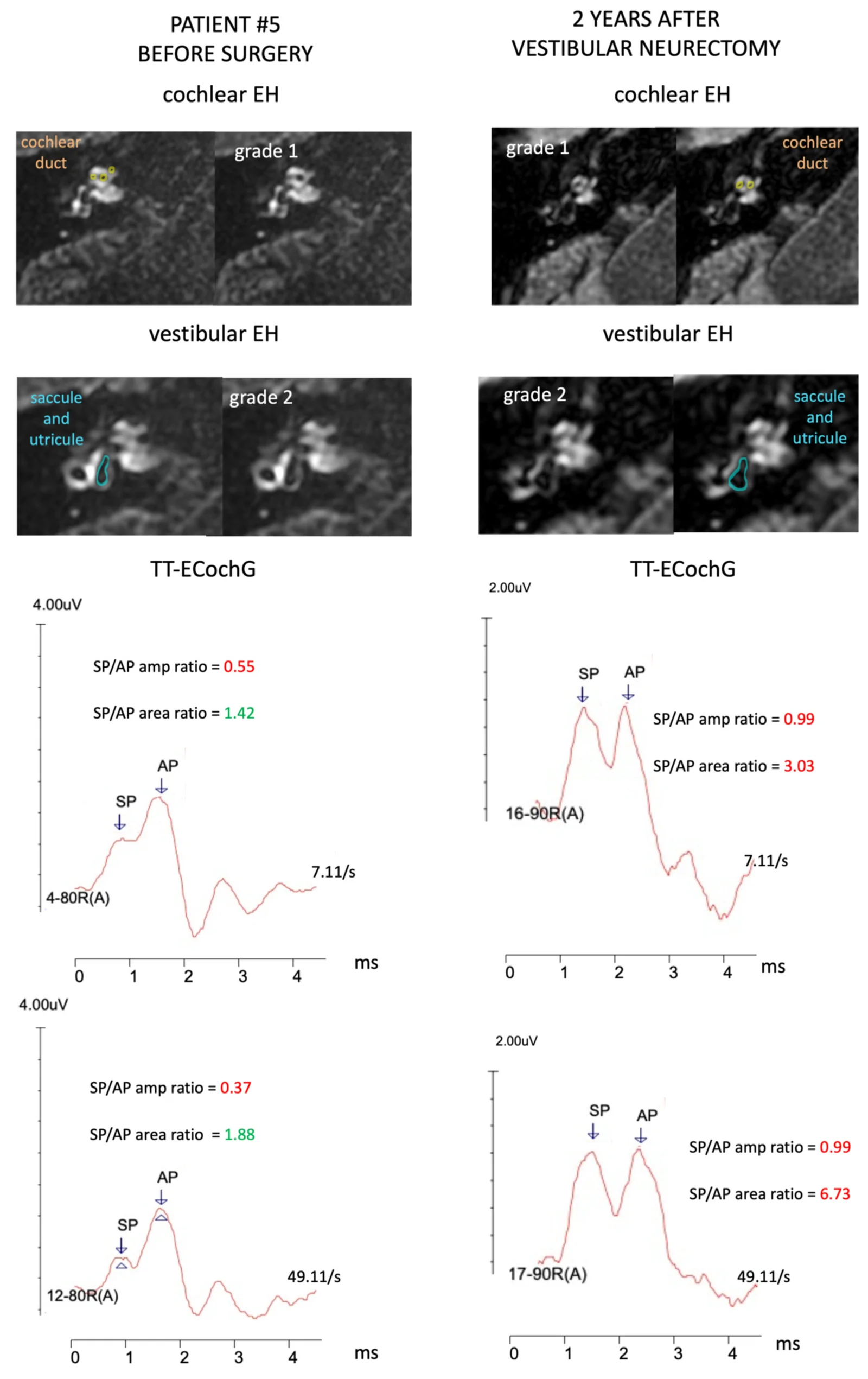
Representative changes in delayed gadolinium-enhanced MRI and transtympanic electrocochleography (TT-ECochG) following vestibular neurectomy. Individual results (patient #5) are shown before surgery (**left column**) and 2 years after vestibular neurectomy (**right column**). Upper panels demonstrate stable cochlear endolymphatic hydrops (cEH grade 1, Barath scale) and vestibular endolymphatic hydrops (vEH grade 2, Bernaerts scale). To facilitate interpretation, line art of the endolymphatic structures is provided with the corresponding MRI images. The lower panels present TT-ECochG recordings obtained at stimulation rates of 7.11/s and 49.11/s. Abnormal results are highlighted in red and normal results in green. Following surgery, the SP/AP amplitude ratio at 7.11/s and both the SP/AP amplitude and area ratios at 49.11/s increased.

Pre- and postoperative audiological and radiological results are presented in Table 2 and Figure 1, Figure 2, Figure 3 and Figure S1.

In four out of five patients, pure-tone audiometry performed during diagnostic hospitalization revealed pantonal sensorineural hearing loss. In the remaining patient (#5), the PTA level was normal; however, hearing thresholds for low-frequency tones were increased. The PTA levels in the affected ears ranged from 17.5 to 70 dB HL (mean, 49.25 dB HL).

TT-ECochG results showed an abnormal SP/AP amplitude ratio (>0.33) at a stimulation rate of 7.11/s in two of five patients (40%), with one of these patients demonstrating a value exceeding the threshold of 0.50. Analyzing SP/AP area ratio results were within the normal range. At 49.11/s, an abnormal SP/AP amplitude ratio was detected in three patients (60%). At 49.11/s, abnormal SP/AP amplitude ratios were detected in three patients (60%). Two of these patients had values above 0.50, and both demonstrated an abnormal SP/AP area ratio (>1.98). When the TT-ECochG result was classified as positive based on at least one abnormal parameter, SP/AP amplitude ratio or SP/AP area ratio at either stimulation rate, pathological preoperative findings were observed in three of five patients (60%) (Table 2).

### 3.5. Postoperative Audiological Results

A significant progression of hearing loss, defined as deterioration of more than 10 dB in the PTA level, was found in two patients (40%) (Figure 1A, Table 2). The average postoperative PTA level in the affected ears was 63.8 dB HL (range, 41.3–88.8 dB HL).

Postoperative TT-ECochG recordings were available in four of five patients, as no response (NR) could be obtained in patient #1 because of profound postoperative hearing loss. At a stimulation rate of 7.11/s, abnormal SP/AP amplitude and area ratios were observed in one patient (#5) (Figure 3). The remaining three evaluable patients had values within the normal range. At 49.11/s, abnormal SP/AP amplitude ratios (>0.33) were detected in three patients (#3, #4, and #5), including two patients (#3 and #5) with values above 0.50 (Figure 2). These same two patients also demonstrated abnormal SP/AP area ratios (>1.98).

Individual postoperative TT-ECochG dynamics were heterogeneous (Figure 1E–H, Table 2). Overall decreases in SP/AP ratios were observed in patients #2 and #4, whereas increases were observed in patients #3 and #5.

### 3.6. Preoperative MRI Results

Both cochlear and vestibular EH were visualized in all patients before surgery (Table 2). Cochlear hydrops was graded as Baráth grade 1 in three patients and grade 2 in two patients. In two patients (40%), moderate vestibular hydrops (Bernaerts grade 2) was present, whereas three patients (60%) demonstrated grade 3 hydrops.

### 3.7. Postoperative MRI Results

At the 2-year follow-up, cochlear hydrops remained unchanged in all patients (Figure 1C, Table 2). Vestibular hydrops improved in two patients (40%), decreasing from Bernaerts grade 3 to grade 2 in patient #3 and from grade 3 to grade 1 in patient #4 (Figure 1D and Figure 2, Table 2), whereas postoperative vestibular hydrops could not be reliably assessed in patient #1 because intense perilymphatic enhancement obscured the vestibule (not analyzed, NA). No patient demonstrated progression of vestibular hydrops after vestibular neurectomy.

## 4. Discussion

Vestibular neurectomy is known to be an effective therapy for uncontrollable MD with vertigo control in over 90% of cases [33,34,35,36,37,38,39,40]. Similarly, all patients described in our pilot study achieved complete resolution of MD attacks after the surgery.

Vestibular neurectomy via the middle fossa approach enables selective section of the vestibular nerve with preservation of cochlear function; however, in a longer observation, hearing deterioration is described in 40–48% of patients [33,39,40], which is consistent with our results.

During neurotological surgeries, i.e., cerebellopontine angle tumor removal or vestibular neurectomy, hearing monitoring may provide additional information about auditory nerve integrity. In addition to TT-ECochG, auditory brainstem responses (ABRs) and direct cochlear nerve action potential (CNAP) recording can be used for real-time assessment [41,42]. Moreover, intracochlear stimulation using the Auditory Nerve Test System (ANTS) has been applied intraoperatively in patients with vestibular schwannoma. Interestingly, transtympanic extracochlear electrical stimulation combined with EABR has been shown to provide information on auditory pathway function, with positive responses associated with auditory benefit after implantation in borderline cochlear implant (CI) candidates [43]. Advanced hearing monitoring may complement conventional audiological tests in future studies, particularly when intraoperative assessment of cochlear nerve integrity and potential CI candidacy is required.

Previous studies evaluating ECochG after vestibular neurectomy were limited to intraoperative or early postoperative examinations. In 1984, Silverstein et al. [44] described intraoperative hearing monitoring using ECochG during retrolabyrinthine vestibular neurectomy. Their study proved that ECochG could provide real-time assessment of cochlear function and help predict postoperative hearing preservation. Moreover, changes in ECochG during vestibular neurectomy via the retrosigmoid approach were analyzed [45]. The authors reported elevated preoperative SP/AP ratios (>0.30) in 79% of patients, with a significant intraoperative reduction observed in 93% after vestibular nerve section. However, reassessment of the cochlear potentials was performed only minutes after surgery, reflecting the immediate physiological effects of vestibular denervation rather than long-term cochlear functional changes. In contrast, long-term electrophysiological outcomes following vestibular neurectomy were evaluated by Kubo et al. [31]. The authors observed a tendency toward increased SP/AP ratios after surgery and presumed that this phenomenon might be related to the olivocochlear bundle manipulation during surgery.

In the present study, the vestibular nerve was sectioned within the internal auditory canal, with removal of the vestibular ganglion to prevent postoperative reinnervation. This technique interrupts the efferent olivocochlear fibers within the vestibular nerve before they reach the cochlea distal to the ganglion via the anastomosis of Oort, an anatomical connection reported in approximately 70% of temporal bone specimens [46]. This may cause a direct cochlear effect of the surgical procedure and result in hearing deterioration. Disruption of the efferent pathway may influence cochlear electrophysiology, but may also have implications for the pathophysiology of endolymphatic hydrops in longer follow-up [47].

ECochG may be useful for monitoring intratympanic treatment response as well. One month after intratympanic dexamethasone therapy, Matrin-Sans et al. [48] observed a significant reduction in the SP/AP ratio associated with improved vertigo control. Nevertheless, another study from the same center demonstrated that the reduction in the SP/AP ratio observed one month after intratympanic dexamethasone treatment was transient [49]. Although a significant decrease was detected shortly after therapy, SP/AP ratios returned to baseline values at the 1-year follow-up despite satisfactory vertigo control in a subset of patients. In patients with refractory MD despite conservative treatment and intratympanic steroids, vestibuloablative intratympanic therapy may be indicated. Gentamicin is known to have a more pronounced vestibulotoxic than cochleotoxic effect; however, the potential risk of hearing loss should always be considered. Recently, worse outcomes of cochlear implantation have been reported in patients previously treated with intratympanic gentamicin [50]. Fiorino et al. [13] analyzed eight patients with MD and reported that EH remained unchanged in four patients and worsened in the remaining four following intratympanic gentamicin treatment. Analyzing results of the intratympanic gentamicin therapy, Büki et al. [51] reported that successful low-dose intratympanic gentamicin therapy resulted in complete vertigo control without significant changes in SP/AP ratios. On the other hand, Adamonis et al. [52] presented a significant reduction in the SP/AP ratio following intratympanic gentamicin therapy. However, these conflicting observations and proposed pathophysiological mechanisms are still speculative, as none of these studies directly assessed endolymphatic hydrops using in vivo MRI.

In the present study, postoperative changes in TT-ECochG tended to parallel hearing outcomes in most patients. Decreased SP/AP ratios accompanied stable hearing thresholds in patients #2 and #4, whereas progressive hearing loss in patient #5 was associated with marked increases in both SP/AP amplitude and area ratios. In contrast, patient #3 showed marked deterioration of TT-ECochG findings despite stable hearing, with PTA increasing by only 8.75 dB. Whereas all preoperative TT-ECochG parameters were within the normal range, three of the four postoperative parameters became abnormal.

Our study evaluates long-term follow-up, analyzing TT-ECochG and EH degree dynamics, providing an evaluation of electrophysiological and morphological changes after vestibular neurectomy. When interpreting our preliminary findings, it is important to recognize that the dynamics of postoperative endolymphatic hydrops following vestibular neurectomy have been evaluated in only a few previous studies [18,19].

Interestingly, in the present study, electrophysiological changes occurred despite stable cochlear EH in all patients. The heterogeneity of TT-ECochG findings confirms that this method should not be considered a stand-alone diagnostic test in MD. However, the dissociation between electrophysiological changes and in vivo assessment of EH suggests that postoperative dynamics in cochlear function may be independent of morphological endolymphatic space dilatation.

Analyzing changes in vestibular EH, postoperative morphological improvement was visualized in patients #3 and #4. Interestingly, patient #4 demonstrated a decrease in both SP/AP amplitude and area ratios, including normalization of the amplitude ratio at a stimulus presentation rate of 7.11/s and the area ratio at a stimulus presentation rate of 49.11/s, while postoperative TT-ECochG of patient #3 became abnormal, despite preoperative results within the normal range. Thus, the observed TT-ECochG changes did not parallel postoperative changes in vestibular EH degree, suggesting that long-term cochlear electrophysiological alterations after vestibular neurectomy cannot be explained solely by changes in the EH severity.

Notably, analyzing results of patient #1, who developed profound postoperative hearing loss and had no measurable TT-ECochG response, postoperative perilymphatic enhancement within the vestibule precluded reliable assessment of vestibular EH. This observation may indicate that postoperative blood–labyrinth barrier alterations could also coincide with electrophysiological findings. Nevertheless, this observation was limited to a single patient and should therefore be considered an incidental finding rather than evidence of a causal relationship. Larger prospective studies are necessary to verify if this finding has any pathophysiological significance.

This study has several limitations. First, the study cohort was small. Vestibular neurectomy is currently reserved for a highly selected group of patients with medically refractory MD and is performed in only a limited number of specialized centers. In addition, TT-ECochG is an invasive procedure; only five of the twenty eligible patients consented to postoperative repeat examination. Therefore, the present study should be regarded as a pilot study. Second, postoperative TT-ECochG and vestibular EH degree could not be evaluated in one patient. Still, we decided to present these results as they may provide clinically relevant observations. Finally, because of the limited sample size, the analyses were primarily descriptive, and the observed associations should be considered hypothesis-generating rather than confirmatory.

## 5. Conclusions

To our knowledge, this is the first study to analyze long-term TT-ECochG, delayed gadolinium-enhanced MRI, and hearing outcomes following vestibular neurectomy. This pilot study shows that postoperative TT-ECochG changes after vestibular neurectomy are heterogeneous and do not consistently parallel MRI changes in endolymphatic hydrops. While cochlear EH remained unchanged in all patients, regression of vestibular EH was observed only in a subset of patients and was not uniformly associated with improvement in electrophysiological parameters. The results of the present study are consistent with the hypothesis that endolymphatic space dilatation is not the sole pathophysiological mechanism underlying MD.

Although preliminary, our findings provide new insight into the relationship between morphological and electrophysiological changes in the inner ear. We hope that these observations will serve as a starting point for further discussion on the pathophysiology of postoperative inner ear changes and stimulate future prospective clinical studies as well as experimental research.

## Figures and Tables

**Table 1:** Preoperative clinical characteristics of the analyzed patients with unilateral definite Ménière’s disease.

No.	Sex	Age (Years)	Affected Ear	Disease Duration (Years)	Vertigo Attacks/Month	AAO-HNS Functional Level	Tumarkin Attacks
**#1**	F	65	Left	2	5.33	5	No
**#2**	F	62	Left	6	4.50	5	No
**#3**	F	35	Right	18	3.00	5	Yes
**#4**	F	56	Right	1	6.33	5	No
**#5**	M	37	Right	1	0.33	4	No

F: female; M: male; AAO-HNS: American Academy of Otolaryngology-Head and Neck Surgery.

**Table 2:** Audiological and MRI findings before and 2 years after middle fossa vestibular neurectomy in patients with unilateral definite Ménière’s disease.

No.	PTA (dB HL)	WRS (%)	cEH Grade	vEH Grade	SP/AP Amp 7.11/s	SP/AP Area 7.11/s	SP/AP Amp 49.11/s	SP/AP Area 49.11/s
	Pre → Post	Pre → Post	Pre → Post	Pre → Post	Pre → Post	Pre → Post	Pre → Post	Pre → Post
**#1**	68.75 → 88.75	45 → 25	2 → 2	2 → NA	0.13 → NR	0.82 → NR	0.21 → NR	1.05 → NR
**#2**	57.50 → 60.00	90 → 70	2 → 2	3 → 3	0.30 → 0.16	1.11 → 0.82	0.72 → 0.33	2.28 → 1.36
**#3**	32.50 → 41.25	100 → 100	1 → 1	3 → 2	0.17 → 0.33	0.74 → 1.15	0.24 → 0.70	1.12 → 2.09
**#4**	70.00 → 78.75	90 → 80	2 → 2	3 → 1	0.47 → 0.28	1.09 → 1.11	1.15 → 0.40	2.01 → 1.31
**#5**	17.50 → 50.00	100 → 100	1 → 1	2 → 2	0.55 → 0.99	1.42 → 3.03	0.37 → 0.99	1.88 → 6.73

PTA—pure-tone average (500, 1000, 2000, and 4000 Hz); dB HL—decibel hearing level; WRS—word recognition score; MRI—magnetic resonance imaging; cEH—cochlear endolymphatic hydrops; vEH—vestibular endolymphatic hydrops; SP—summating potential; AP—action potential; Amp—amplitude; NR—not recorded; NA—not analyzed.

## Data Availability

The dataset is available on request from the authors.

## Supplementary Materials

The following supporting information can be downloaded at: https://www.mdpi.com/article/10.3390/jcm15187164/s1, Figure S1: Pre- and postoperative results of delayed gadolinium-enhanced MRI and transtympanic electrocochleography (TT-ECochG). Individual results of cochlear and vestibular endolymphatic hydrops (EH) in Patients #1, #2, and #3 are shown in panels A, B, and C, respectively. The lower panels present TT-ECochG recordings obtained at stimulation rates of 7.11/s and 49.11/s. Abnormal results are highlighted in red and normal results in green.

## Abbreviations

The following abbreviations are used in this manuscript:

MD	Ménière’s disease
MRI	Magnetic resonance imaging
AAO-HNS	American Academy of Otolaryngology-Head and Neck Surgery
EH	Endolymphatic hydrops
TT-ECochG	Transtympanic electrocochleography
SP	Summating potential
AP	Action potential
