# Supplementary material for: Do Changes in Transtympanic Electrocochleography (TT-ECochG) Reflect the Dynamics of Endolymphatic Hydrops on MRI? Audiological and Radiological Longitudinal Analysis in Patients with Severe Ménière’s Disease After Vestibular Neurectomy—A Pilot Study
Source: J Clin Med. 2026 Sep 15;15(18):7164. doi: 10.3390/jcm15187164 (PMC13607096; doi:10.3390/jcm15187164)
Supplement: Supplementary file 1 [file jcm-15-07164-s001.zip › jcm-4531942-supplementary.pdf]

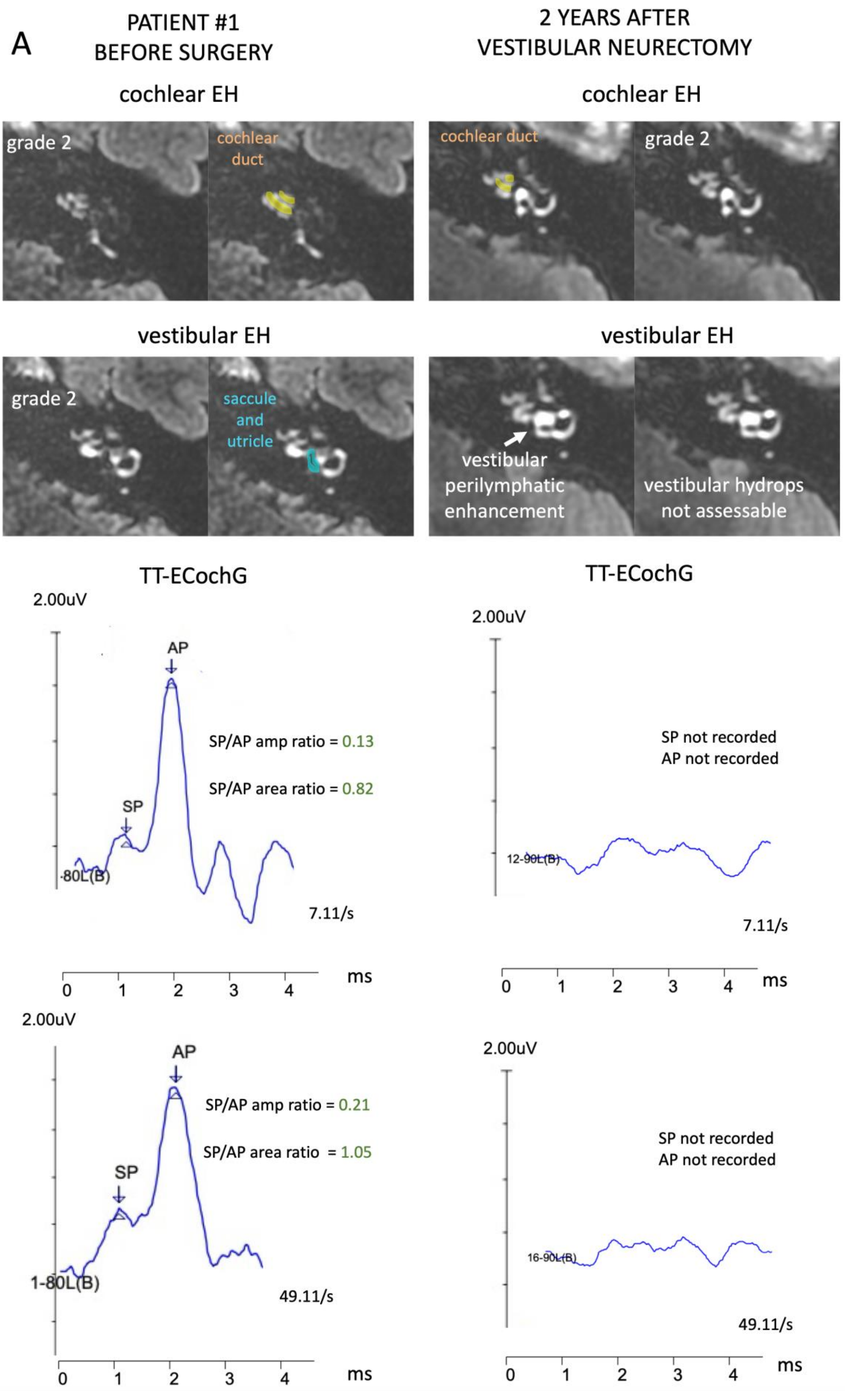
A
PATIENT #1
BEFORE SURGERY
2 YEARS AFTER
VESTIBULAR NEURECTOMY
cochlear EH
grade 2
cochlear duct
cochlear EH
cochlear duct
grade 2
vestibular EH
grade 2
saccule and utricle
vestibular EH
vestibular perilymphatic enhancement
vestibular hydrops not assessable
TT-ECochG
2.00uV
AP
SP
SP/AP amp ratio = 0.13
SP/AP area ratio = 0.82
80L(B)
7.11/s
0 1 2 3 4 ms
TT-ECochG
2.00uV
SP not recorded
AP not recorded
12-90L(B)
7.11/s
0 1 2 3 4 ms
2.00uV
AP
SP
SP/AP amp ratio = 0.21
SP/AP area ratio = 1.05
1-80L(B)
49.11/s
0 1 2 3 4 ms
2.00uV
SP not recorded
AP not recorded
16-90L(B)
49.11/s
0 1 2 3 4 ms

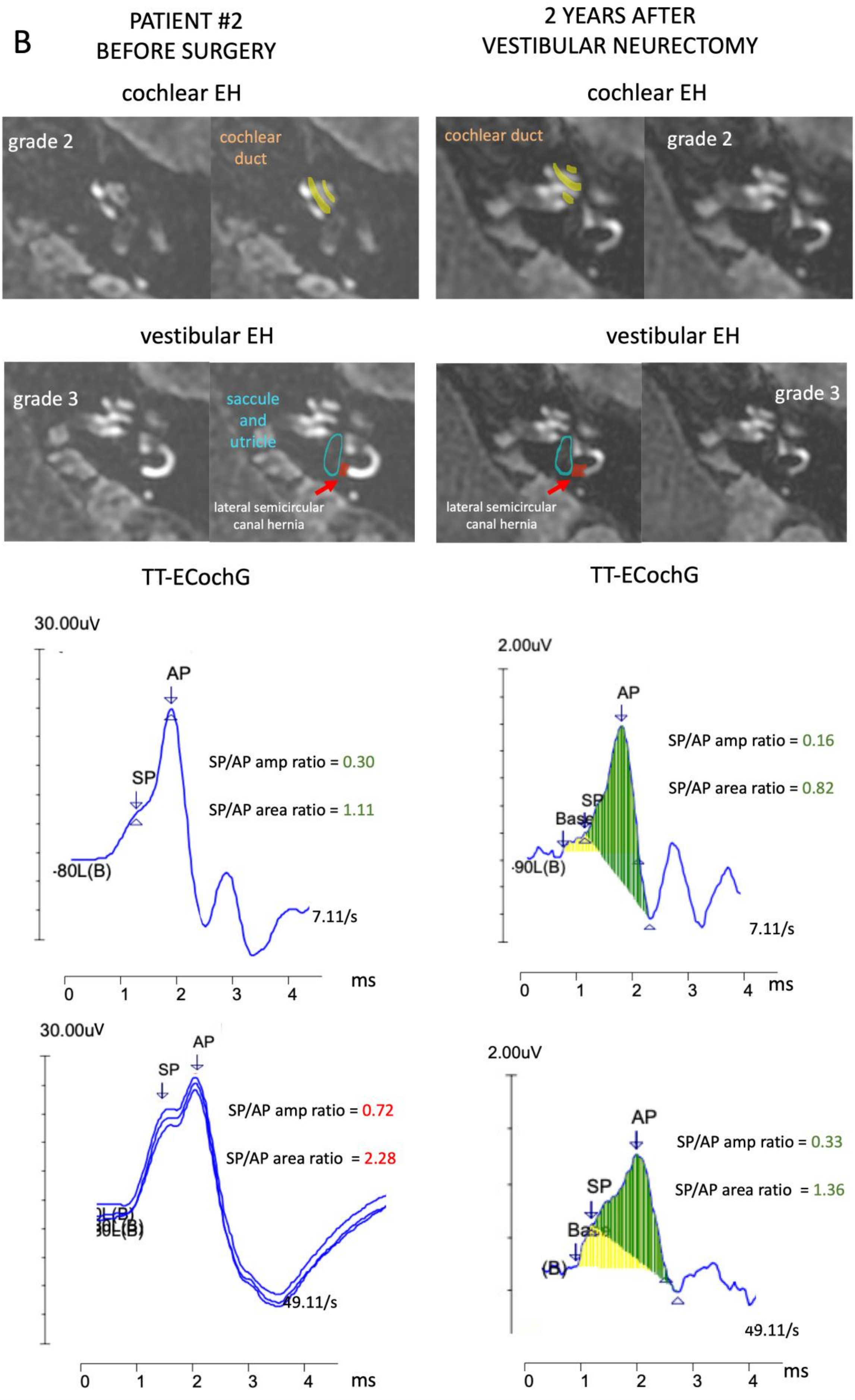
B
PATIENT #2
BEFORE SURGERY
2 YEARS AFTER
VESTIBULAR NEURECTOMY
cochlear EH
grade 2
cochlear duct
cochlear EH
cochlear duct
grade 2
vestibular EH
grade 3
saccule and utricle
lateral semicircular canal hernia
vestibular EH
lateral semicircular canal hernia
grade 3
TT-ECochG
30.00uV
AP
SP
SP/AP amp ratio = 0.30
SP/AP area ratio = 1.11
-80L(B)
7.11/s
ms
0 1 2 3 4
TT-ECochG
2.00uV
AP
SP
Base
SP/AP amp ratio = 0.16
SP/AP area ratio = 0.82
-90L(B)
7.11/s
ms
0 1 2 3 4
30.00uV
AP
SP
SP/AP amp ratio = 0.72
SP/AP area ratio = 2.28
49.11/s
ms
0 1 2 3 4
2.00uV
AP
SP
Base
(B)
SP/AP amp ratio = 0.33
SP/AP area ratio = 1.36
49.11/s
ms
0 1 2 3 4

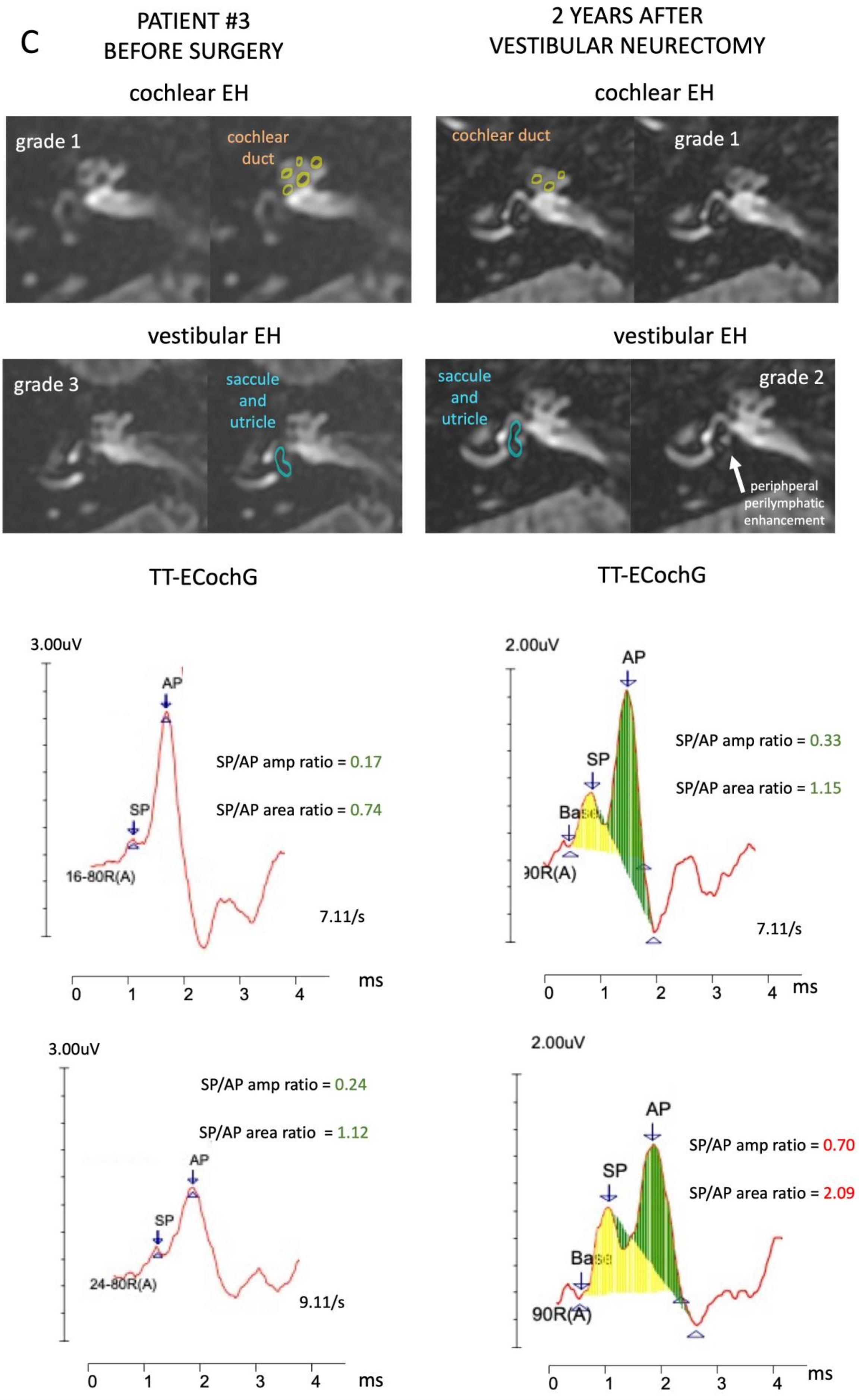
C
PATIENT #3
BEFORE SURGERY
2 YEARS AFTER
VESTIBULAR NEURECTOMY
cochlear EH
grade 1
cochlear duct
cochlear EH
cochlear duct
grade 1
vestibular EH
grade 3
saccule and utricle
vestibular EH
saccule and utricle
grade 2
periphperal perilymphatic enhancement
TT-ECochG
TT-ECochG
3.00uV
AP
SP
16-80R(A)
SP/AP amp ratio = 0.17
SP/AP area ratio = 0.74
7.11/s
0 1 2 3 4 ms
2.00uV
AP
SP
Base
90R(A)
SP/AP amp ratio = 0.33
SP/AP area ratio = 1.15
7.11/s
0 1 2 3 4 ms
3.00uV
SP/AP amp ratio = 0.24
SP/AP area ratio = 1.12
AP
SP
24-80R(A)
9.11/s
0 1 2 3 4 ms
2.00uV
AP
SP
Base
90R(A)
SP/AP amp ratio = 0.70
SP/AP area ratio = 2.09
0 1 2 3 4 ms

Figure S1. Pre- and postoperative results of delayed gadolinium-enhanced MRI and transtympanic electrocochleography (TT-ECochG). Individual results of cochlear and vestibular endolymphatic hydrops (EH) in Patients #1, #2, and #3 are shown in panels A, B, and C, respectively. The lower panels present TT-ECochG recordings obtained at stimulation rates of 7.11/s and 49.11/s. Abnormal results are highlighted in red and normal results in green.
